# EHMTI-0038. Does hypophysial-gonadal system influence formation of tension-type headache in women of reproductive age?

**DOI:** 10.1186/1129-2377-15-S1-F5

**Published:** 2014-09-18

**Authors:** K Derevyanko

**Affiliations:** 1 Neurology, Clinic of Bashkir State Medical University, Ufa, Russia

## 

The obvious difference in pain prevalence related to gender is well known, but the underlying mechanisms are not clearly identified. Female sex steroids may play an important role in this difference.

## Objective

Estimation of hypophysial-gonadal hormones level and psychoemotional status in women of reproductive age with tension-type headache

## Methods

We have evaluated the content of hypophysial-gonadal hormones in blood serum in luteal and follicular phases. Diagnosis has been made using ICHD-II. Psychoemotional status has been assessed by the scale of Beck, Hamilton and Spilberg.

## Results

1.At moderate personal and reactive anxiety and depression in women with chronic tension-type headache (CTTH) the increased cortisol in blood has been found.

2. In a follicular phase the women with CTTH had the increased luteinizing (52.2±7.5 IU/l) and FSH (18.2±1.4 IU/l), while in a luteal phase- progesterone was high (89.7 ±3.6 nmol/l). Estradiol (274.3±25.3 pg/ml), testosterone (2.4±0.3ng/ml) and cortisol (877.2±35.6 nmol/l) were increased as in the luteal and follicular phases of the ovary cycle. Quantitatively, the hypothesis-ovary and hypothesis-adrenal cortex hormones in patients with episodic tension headache were within the normal range in both phases of the ovary cycle.

3. In women CTTH was accompanied by moderate reactive and personal anxiety and evident depression during the ovary cycle becoming more intensive. The women with episodic tension-type headache demonstrated low anxiety and absence of depression in follicle and luteinizing phases.

## Conclusion

The obtained data allow us to trace the pathogenic correlation between CTTH and hormonal deviations

No conflict of interest.

